# KSHV Entry and Trafficking in Target Cells—Hijacking of Cell Signal Pathways, Actin and Membrane Dynamics

**DOI:** 10.3390/v8110305

**Published:** 2016-11-14

**Authors:** Binod Kumar, Bala Chandran

**Affiliations:** H. M. Bligh Cancer Research Laboratories, Department of Microbiology and Immunology, Chicago Medical School, Rosalind Franklin University of Medicine and Science, North Chicago, IL 60064, USA; binod.kumar@rosalindfranklin.edu

**Keywords:** KSHV entry, integrins and EphA2R receptors, macropinocytosis, clathrin endocytosis, signal induction, KSHV trafficking

## Abstract

Kaposi’s sarcoma associated herpesvirus (KSHV) is etiologically associated with human endothelial cell hyperplastic Kaposi’s sarcoma and B-cell primary effusion lymphoma. KSHV infection of adherent endothelial and fibroblast cells are used as in vitro models for infection and KSHV enters these cells by host membrane bleb and actin mediated macropinocytosis or clathrin endocytosis pathways, respectively. Infection in endothelial and fibroblast cells is initiated by the interactions between multiple viral envelope glycoproteins and cell surface associated heparan sulfate (HS), integrins (α3β1, αVβ3 and αVβ5), and EphA2 receptor tyrosine kinase (EphA2R). This review summarizes the accumulated studies demonstrating that KSHV manipulates the host signal pathways to enter and traffic in the cytoplasm of the target cells, to deliver the viral genome into the nucleus, and initiate viral gene expression. KSHV interactions with the cell surface receptors is the key platform for the manipulations of host signal pathways which results in the simultaneous induction of FAK, Src, PI3-K, Rho-GTPase, ROS, Dia-2, PKC ζ, c-Cbl, CIB1, Crk, p130Cas and GEF-C3G signal and adaptor molecules that play critical roles in the modulation of membrane and actin dynamics, and in the various steps of the early stages of infection such as entry and trafficking towards the nucleus. The Endosomal Sorting Complexes Required for Transport (ESCRT) proteins are also recruited to assist in viral entry and trafficking. In addition, KSHV interactions with the cell surface receptors also induces the host transcription factors NF-κB, ERK1/2, and Nrf2 early during infection to initiate and modulate viral and host gene expression. Nuclear delivery of the viral dsDNA genome is immediately followed by the host innate responses such as the DNA damage response (DDR), inflammasome and interferon responses. Overall, these studies form the initial framework for further studies of simultaneous targeting of KSHV glycoproteins, host receptor, signal molecules and trafficking machinery that would lead into novel therapeutic methods to prevent KSHV infection of target cells and consequently the associated malignancies.

## 1. Introduction

To control and/or eliminate any viral infection causing a spectrum of human and animal diseases by vaccines and/or anti-viral agents, comprehensive information regarding the replication, biology, and pathogenesis of the causative viral agent is essential. Foremost in this requirement is the thorough understanding of the complex multistep process of virus entry into target cells. To establish a successful infection, viruses need to rapidly counteract a number of hindrances in the host cells, such as the barrier of plasma membrane, underlying actin cytoskeleton, dense packed cytoplasm, induction of intrinsic and innate cellular protective immune responses including apoptosis, autophagy, DNA damage responses (DDR), and restriction on viral gene transcription and translation. Viruses not only depend on their gene products to overcome these obstacles but have also evolved to hijack host cell molecules including the host cell’s preexisting signal pathways to tackle these hindrances and to create a favorable milieu that facilitates the establishment of infection.

More than 100 herpesviruses of the family Herpesviridae infect many different species across the animal kingdom and have evolved in parallel with their respective hosts for millions of years [1]. Herpesviruses are characterized by their complex linear double stranded DNA genome (~110–230 Kb) that is enclosed in an icosahedral capsid of ~100–125 nm, surrounding layers of several tegument proteins, which in turn is enclosed in a double layered membrane (envelope) bearing several virally coded glycoproteins. An important attribute of all herpesviruses is that after primary infection and lytic replication in the host cell resulting in progeny virion formation, all of them establish a latent infection in several specific host cells with limited latent gene expression. Periodic reactivation of the latent state results in reinfection of new cells with the continuation of latent infection.

Herpesviruses are classified as α-, β- and γ-subfamilies of herpesviruses based on the cell tropism and genome similarities [2]. The α-subfamily includes the human herpes simplex virus type-1 and 2 (HSV-1 and HSV-2) and varicella-zoster virus (VZV). The β-subfamily consists of the human cytomegalovirus (HCMV), HHV-6 variants A and B, and HHV-7, while the γ-subfamily consists of the Epstein–Barr virus (EBV) and the Kaposi’s sarcoma-associated herpesvirus (KSHV) or human herpesvirus-8 (HHV-8). Most humans are infected with HSV-1, EBV, VZV, CMV, HHV-6, and HHV-7 during early life. HSV-2 is predominately acquired during the sexually active phase of life. KSHV is believed to be transmitted through saliva in young siblings in some parts of the world such as French Guiana [3] or sub-Saharan Africa [4] where the disease is endemic, and through sexual contacts in risk groups such as homosexual men in low prevalence countries [5]. Reactivation of these viruses under immunocompromised conditions results in severe life-threatening diseases including neoplasms by EBV and KSHV.

Target cell infection by herpesviruses is initiated by the binding and interaction of viral envelope glycoproteins with several cell surface molecules (receptors). These interactions lead into either fusion of the viral envelope with the host cell membrane or entry of enveloped viral particles via endocytosis and subsequent fusion of the viral envelope with the endocytic membrane resulting in the release of viral dsDNA containing capsid surrounded by a number of tegument proteins, and potentially a number of viral and host microRNAs (miRNA) and long non-coding RNA (lncRNA). Cytoplasmic capsid is rapidly transported towards the nuclear periphery by the dynein motors via the microtubules and the capsid is disassembled near the nuclear pore by a mechanism not fully understood resulting in the entry of viral DNA into the nucleus.

### Kaposi’s Sarcoma-Associated Herpesvirus (KSHV) or Human Herpesvirus-8 (HHV-8)

KSHV, a member of the γ2-lymphotropic-oncogenic herpesviruses, is implicated in the etiology of several human cancers such as Kaposi’s sarcoma (KS) [6,7], primary effusion B-cell lymphoma (PEL) or body-cavity based B-cell lymphoma (BCBL) [8], and B-cell proliferative multicentric Castleman’s disease [9,10]. KSHV has also been shown to be the cause of KSHV inflammatory cytokine syndrome (KICS) [11,12]. KSHV’s ~160 Kbp DNA genome [13,14] is closely related to γ-1 EBV, and the γ-2 herpesvirus saimiri (HVS) and Rhesus monkey rhadinovirus (RRV) [15]. KSHV encodes more than 100 open reading frames (ORF), 17 viral microRNAs (vmiRNA) and several viral long non-coding RNAs (vlncRNA), and ORFs 4–75 are named by their homology with HVS ORFs [16,17]. The KSHV genome consists of three gene blocks that are conserved with other herpesviruses as well as gene blocks encoding more than 20 genes unique to KSHV, which are named K genes.

KSHV has a broad cellular tropism as it infects a variety of in vivo target cells such as endothelial cells, B cells, monocytes, epithelial cells and keratinocytes, and establishes latency [10]. Although B cells are a major reservoir for KSHV persistence in the body, these B cells are extremely difficult to infect with KSHV in the laboratory, thus posing a hindrance to studies on B cell infection. In a recent study, it was observed that MC116, an EBV negative American Burkitt’s lymphoma B cell line, can be infected by cell-free recombinant strain KSHV.219 (rKSHV.219) resulting in latency [18]. A study has also shown that KSHV can infect human primary tonsillar B cells and these B cells are non-cytolytically controlled by CD4^+^ T cells [19]. KSHV also infects human and mouse fibroblast cells, owl monkey kidney cells, BHK-21 (baby hamster kidney) cells and CHO (Chinese hamster ovary) cells [20,21]. However, KSHV does not infect the rodents in vivo and hence there are no small or primate animal models that will faithfully emulate human KSHV infection and pathogenesis.

Studies have shown that KSHV utilizes its multiple glycoproteins to bind to the host cell membrane followed by interaction with specific entry receptors to induce a cascade of signal pathways to promote endocytosis. KSHV enters the cells in a cell type specific manner, and recent studies demonstrate that it hijacks the host cellular ESCRT machinery (endosomal sorting complexes required for transport) for the enveloped virion containing endosome trafficking and release of capsid near the nuclear pore area resulting in the delivery of viral DNA into the nucleus. All these events collectively also lead to the simultaneous induction of the host innate immune responses against the invading pathogen.

In this review, we summarize the studies available to date to portray the various stages of the complex pathways of KSHV entry, trafficking and delivery of viral genome into the nucleus early during infection of human microvascular dermal endothelial cells (HMVEC-d) and human foreskin fibroblast (HFF) cells. We have given emphasis to: (a) different endocytic entry pathways of KSHV; (b) KSHV envelope glycoproteins and their interactions with the host cell surface associated receptors and lipid rafts (LR); (c) resulting induction of pre-existing host cell signal pathways; and (d) the influence of the induced signal pathways on the: (i) consequent membrane and actin dynamics; (ii) intracellular KSHV traffic utilizing the host cell membranes and actin; and (iii) recruitment of trafficking machinery and their role in KSHV infection. As virus entry and infection is intimately associated with the host innate cellular responses, we also discuss briefly the responses elicited during the early stages of KSHV entry and recognition of the viral genome.

## 2. KSHV Utilizes Diverse Endocytic Pathways for Entry into the Target Cells

KSHV enters human B cells, fibroblast, epithelial, and endothelial cells by endocytosis and utilizes macropinocytosis and clathrin-mediated endocytosis to enter HMVEC-d and HFF cells, respectively [22,23,24,25,26]. Enveloped viral particles are detected inside the endocytic vesicles as early as 5 min post-infection (p.i.) in HMVEC-d and HFF cells which penetrate through the endocytic membranes in an acid pH-dependent manner to enter the cytosol, and the capsid then reaches near the nuclear periphery within 15 min p.i. via the host microtubules [24]. Apart from KSHV, HSV-1 has also been shown to utilize macropinocytosis as the mode of entry in some of the target cells [27,28].

### 2.1. Macropinocytic Mode of Entry

**Figure 1:**
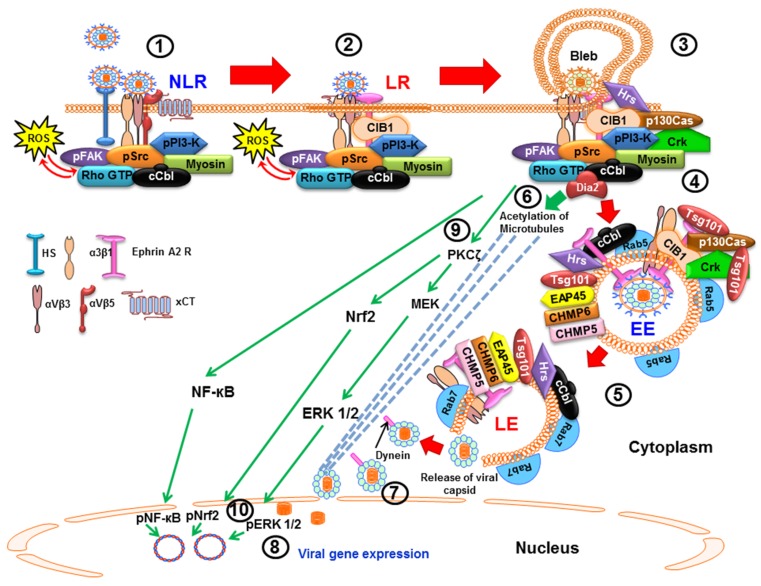
Schematic diagram depicting the entry of KSHV by macropinocytosis and subsequent trafficking in human microvascular dermal endothelial cells (HMVEC-d) and the various signal cascades induced by KSHV early during infection to aid in the various steps of infection. (1) The initial attachment of KSHV is with HS in the non-lipid raft (NLR) region of the membranes which concentrates the virus particles. This is immediately followed by the interactions with α3β1, αVβ3 and αVβ5 integrins and xCT molecules. (2) Within 1–5 min of infection, this interaction induces the phosphorylation of FAK, Src, PI3-K as well as recruitment of the adaptor proteins c-Cbl and CIB1, c-Cbl mediated monoubiquitination of α3β1 and αVβ3, and rapid translocation of KSHV into the lipid raft (LR) along with the α3β1, αVβ3, and x-CT receptors but not αVβ5. KSHV interactions with the receptors also lead to the production of ROS, which in turn amplifies FAK, Src and the Rho-GTPase and Rac1. (3) In the LR, KSHV interacts with EphA2R, which amplifies the signal cascades. (4) Infection induced ESCRT-0 protein Hrs associates with the membrane and ROCK1 and induces a local pH change. Activated c-Cbl interacts with myosin IIA and results in bleb formation, its retraction and macropinosome formation that enclose the viral particles with virus associated integrins and EphA2R in the luminal face of the vesicles and CIB1, Crk, and p130Cas in the cytoplasmic side. (5) Virus containing early and late endosomes is associated with Rab5 and Rab7, respectively, as well as with integrins, EphA2, CIB1, Crk, p130Cas and ESCRT-I-III proteins. The KSHV lipid envelope membrane fuses with the endosomal membrane mediated by the viral glycoproteins to release the capsid and the enclosed dsDNA viral genome into the cytoplasm. (6) RhoA-GTPase mediated Dia-2 dependent acetylation of microtubules helps in the rapid transport of capsid towards the nucleus and the capsid disassembles near the nuclear pore resulting in the delivery of viral DNA into the nucleus (7). (8) Viral gene expression is initiated by the infection induced host ERK1/2, NF-κB and Nrf2 transcription factors that translocate to the nucleus from the cytoplasm (9 and 10). Studies summarized in this review demonstrate that: (i) KSHV infection induced FAK, Src, PI3-K, Rho-GTPase and ROS play roles in KSHV entry by endocytosis and actin remodeling; (ii) PI3-K, RhoA-GTPase and Dia 2 molecules play roles in microtubule acetylation, reorganization and transport of capsid to the nuclear vicinity; and (iii) ERK1/2, NF-κB and Nrf2 play roles in viral and host gene expression and modulation.

Macropinocytosis, a particular form of endocytosis, is the major route of productive KSHV infection in endothelial HMVEC-d and HUVEC cells (umbilical vein endothelial cells) (Figure 1). The role of macropinocytosis was revealed by studies that used macropinocytosis inhibitors such as 5-(*N*-ethyl-*N*-isopropyl)-amiloride (EIPA-sodium-proton exchange inhibitor) and rottlerin to demonstrate the significant inhibition of both KSHV entry and gene expression [24]. These studies also showed the co-endocytosis of macropinocytosis marker dextran with KSHV, and envelope labeled virus-DiI-KSHV (lipophilic dye, DiI-1,1′-dioctadecyl-3,3,3′,3′-tetramethylindocarbocyanine perchlorate) colocalization with Rab5 and Rab7 during KSHV entry in both endothelial cell types by immunofluorescence microscopy and flow cytometry [24]. These observations were strongly supported by control experiments with the clathrin pathway marker transferrin and KSHV co-endocytosis, wherein no appreciable colocalization was observed in the endothelial cells.

Membrane blebbing is an important event during KSHV macropinocytic entry and use of blebbistatin, a potent inhibitor of membrane blebbing, inhibits the entry of KSHV significantly [29]. Studies also reveal that the LRs direct the clustering of KSHV bound receptors EphA2R and integrins to direct macropinocytosis towards productive infection [30]. The EphA2R also synergizes with the adaptor CIB1 molecule to sustain signal amplification and promote macropinocytosis [31]. A recent study has also demonstrated the macropinocytic route of KSHV entry dependent upon Hepatocyte growth factor-regulated tyrosine kinase substrate (Hrs), an ESCRT-0 component of the ESCRT machinery, which translocates to the plasma membrane near the virus induced blebs and assists in viral entry via Rho-associated protein kinase 1 (ROCK1) mediated phosphorylation of Sodium/hydrogen exchanger 1 (NHE1) and modulates a local pH change essential for macropinocytosis [32]. Another recent finding revealed the role of ESCRT-I protein Tsg101 in KSHV trafficking and transition from early to late endosomes [33], thus defining novel roles of host proteins that are involved during viral entry and infection.

Although several studies have shown that KSHV enters the endothelial cells by macropinocytosis, they all have been performed in tissue culture experiments and their validation in vivo is still a difficult task as there is no animal model for studying KSHV till date.

### 2.2. Clathrin-Mediated Endocytic Mode of Entry

**Figure 2:**
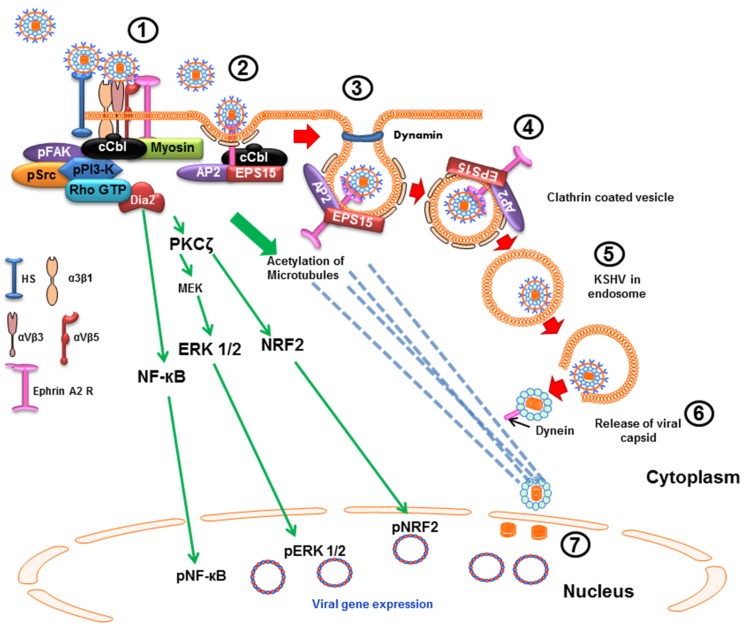
Schematic diagram depicting the clathrin mediated endocytosis of KSHV and trafficking in human foreskin fibroblast (HFF) cells. (1) The initial attachment of KSHV is with HS in the NLR regions of the plasma membrane which is followed by interactions with α3β1, αVβ3, and αVβ5 integrins and with EphA2R. The association of EphA2R with integrins leads to the formation of an active signaling complex which leads to the induction of FAK, Src, and PI3-K signal molecules. (2) PI3-K activated c-Cbl then polyubiquitinates EphA2R and recruits the accessory proteins Eps15 and adaptor protein AP-2, which is followed by (3 and 4) assembly of clathrin, formation of clathrin coated pits, internalization of KSHV into clathrin coated vesicles along with the activated signaling platforms and the associated molecules leading to the dynamin dependent release of the vesicles. (5) The internalized vesicles then recruit Rab5 and Rab7, and the virion envelope membrane fuses with the endosomal membrane mediated by the viral glycoproteins to release the capsid, which is transported (6) near the nuclear pore area resulting in genome entry into the nucleus. As in HMVEC-d cells, (i) KSHV infection induced FAK, Src, PI3-K, Rho-GTPase and ROS play roles in KSHV entry and actin remodeling; (ii) PI3-K, RhoA-GTPase and Dia 2 molecules play roles in microtubule acetylation, reorganization and transport of capsid to the nuclear vicinity; and (iii) ERK1/2, NF-κB and Nrf2 play roles in viral and host gene expression and modulation (7).

Clathrin-mediated endocytosis is another major route involving the uptake of materials into the cell from the surface using clathrin-coated vesicles. It is one of the most common routes of viral internalization and this pathway is utilized by several viruses such as hepatitis C virus [34], Ebola virus [35], influenza A virus, vesicular stomatitis virus, etc. [28]. KSHV enters HFF cells via clathrin mediated endocytosis. Use of chlorpromazine, a clathrin pathway inhibitor, significantly inhibits KSHV entry into HFF cells, whereas the caveolae inhibitor and LR inhibiting agents have no effect on entry [24,25]. KSHV interacts with HFF cell surface HS, integrins (α3β1, αVβ3 and αVβ5), and EphA2R in the non-LR region, and interactions with EphA2R result in the formation of an active signaling complex of integrins, FAK, Src and PI3-K, c-Cbl and myosin IIA which subsequently activates the downstream pathways (Figure 2). Within minutes of infection, KSHV induced PI3-K activates c-Cbl which polyubiquitinates EphA2R and recruits the accessory protein Eps15 and an adaptor protein AP-2 which promote the activation, recruitment and assembly of clathrin resulting in the formation of clathrin coated pits (CCP). These signaling complexes aid in KSHV internalization into clathrin coated vesicles. Subsequently, KSHV infection induced pre-existing host cell signal pathway, as discussed in the later sections here, aids in KSHV trafficking into the early and late endosome, acidification of endosome, nuclear entry of viral DNA and viral gene expression [36] in a manner similar to macropinocytic entry in endothelial cells. KSHV also enters BJAB cells, a KSHV and EBV negative B-cell lymphoma cell, and HEK293 (human embryonic kidney) cells through the clathrin endocytic pathway [22].

## 3. KSHV Infection Is Initiated by Its Binding to Receptors on the Host Cell Membrane

### 3.1. Interactions with Cell Surface Proteins and Viral Entry Are Mediated by KSHV Envelope Glycoproteins

The KSHV envelope glycoproteins play key roles not only in virion binding to the host cell plasma membrane associated molecules (receptors) and subsequent entry during primary target cell infection, but also in the complex process of nuclear assembly of enveloped viral particles, re-envelopment in the cytoplasmic vesicles and egress of the progeny virus during a lytic infection. KSHV envelope has several glycoproteins such as gB (ORF8), gH (ORF22), gL (ORF47), gM (ORF39), and gN (ORF53) that are conserved with other herpesviruses [15,37,38]. It also encodes for some unique lytic cycle associated glycoproteins such as ORF4, gpK8.1A, gpK8.1B, K1, K14 and K15, and among which ORF4 and gpK8.1A are associated with the viral envelope [39,40,41,42].

The major envelope glycoprotein involved in the initiation of virus binding and entry is KSHV gB consisting of disulfide linked polypeptides of 75-kDa and 54-kDa [39,41,43]. KSHV gB mediates viral binding and entry by its interaction with host cell surface HS, and entry receptors α3β1, αVβ3 and αVβ5 integrins in HMVEC-d and HFF cells [41,42,43,44,45]. gB has a high mannose carbohydrate structure and binds to DC-SIGN (Dendritic cell specific intracellular adhesion molecule-3 (ICAM-3) grabbing non-integrin) molecule of B-cells [46]. KSHV-gB interaction with integrins activates the associated signal molecules such as FAK, Src, PI3K, and Rho-GTPase [47]. Apart from its role in virus entry, gB is also shown to be critical for KSHV maturation and egress [48]. The KSHV gpK8.1 gene encodes two alternatively spliced messages yielding glycoproteins gpK8.1A and gpK8.1B, the former being the main form expressed on infected cells and assembled on the virion envelope [37,49,50]. Similar to gB, KSHV gpK8.1A also possesses the ability to bind to the HS molecule [51,52].

Like other herpesviruses, KSHV glycoproteins gH and gL form a non-covalently linked gH/gL complex where gL plays an important role in complex formation by promoting intracellular gH trafficking [53,54,55]. gH and ORF4 have also been shown to interact with HS, and blocking gH/gL with anti-gH and anti-gL antibodies inhibits KSHV entry but not KSHV binding [55]. Recent studies also showed that gH/gL interacts with the KSHV entry receptor EphA2R and are crucial for KSHV entry [54]. The KSHV gM and gN glycoproteins form a heterodimeric complex and are involved in virus penetration and egress. Similar to the gH/gL complex, the KSHV gN is essential for gM’s post-translational modification and trafficking to the cell surface [56].

### 3.2. Heparan Sulfate (HS) Is Utilized by KSHV as the Initial Binding Receptor

KSHV, like other herpesviruses, relies on two types of host cellular receptors, the binding and the entry receptors. HS is an ubiquitously expressed host cell membrane proteoglycan that facilitates charge based KSHV attachment and concentration on the cell surface which subsequently causes conformational changes in the virus glycoproteins to gain access to the adjacent entry receptors. Preincubation of virus with soluble heparin, but not with soluble chondroitin sulfate A and C, results in the inhibition of KSHV binding, subsequent signal induction and infection, thus demonstrating the specific role of HS in KSHV attachment to the cells [41] (Table 1). The Ext1 enzyme required for HS glycosylation is absent in several B cell lines and primary B cells resulting in their refraction to KSHV infection. The role of HS was further strengthened by the results demonstrating KSHV infection in the BJAB cells expressing HS and the absence of infection in the cells lacking HS [21] (Table 1).

KSHV envelope glycoproteins gB, gpK8.1A, ORF4 and gH bind HS to facilitate speedy concentration of KSHV on the cell surface [43,44,51,54]. Pretreatment of KSHV with soluble heparin, enzymatic removal of cell surface HS by heparinase I and III or pretreatment with soluble gB and gpK8.1A inhibits KSHV infection [43,51,52]. The KSHV gB extracellular domain possesses a conserved heparin binding domain (HBD) motif HIFKVRRYRK at amino acids 108–117, and KSHV gpK8.1A has an atypical HBD whereas it is missing in gH [41,55].

### 3.3. Integrins on Endothelial, Fibroblast and Monocyte Cell Surfaces Play a Role as KSHV Entry Receptors

Integrins are a widely expressed family of cell adhesion receptors which mediates a variety of functions such as the attachment of cells to the extracellular matrix, outside to inside signaling events, tissue remodeling, etc. Integrins are utilized for attachment and/or cell entry by several enveloped and non-enveloped viruses. Among all the herpesviruses, studies with KSHV were the first to show the utilization of integrins as entry receptors of herpesviruses in adherent cells [44]. An integrin binding RGD (Arg-Gly-Asp) motif is present at amino acids 27–29 of KSHV envelope glycoprotein gB [44] which is the minimal peptide sequence of many integrin ligands known to interact with a subset of cellular integrins. Studies have established the roles of α3β1, αVβ3 and αVβ5 integrins during KSHV infection in HFF, HMVEC-d, HEK293, Vero cells and monocytes [30,44,45,57,59].

Although some studies do show discrepancies regarding the integrin subtypes used by KSHV in different target cells [22,59], experimental methodologies and differences in the cells used could account for these discrepancies. Studies using RGD peptides and antibodies against RGD-gB peptides have shown the important role of integrins as entry receptors for KSHV. Pretreatment of HMVEC-d, HFF cells, human fibrosarcoma cell line HT-1080 and monkey kidney cell line CV-1 cells with anti-αV and anti-β1 integrin antibodies inhibits the cell attachment mediated by KSHV-gB [44,45,59] (Table 1). Functional blocking anti-α3 and β1 integrin antibodies inhibited ~50% of infection in HMVEC-d and HFF cells. Overexpression of α3 integrin in CHO cells increases the infection; however, as the infection is not as robust as in HMVEC-d and HFF cells, this suggests the participation of other receptors in KSHV infection that are not present in the CHO cells [45]. KSHV attachment sites have been shown to be present in specific cellular microdomains that consist of actin-based filopodia, lamellipodia, ruffled membranes, microvilli, intercellular junctions, and integrins αVβ3, αVβ5 and α3β1 [66]. Interaction of KSHV disintegrin domains of gB with α9β1 integrin is believed to also lead into virus entry in the HFF cells [67]. Although the anti-α9 and β1 integrin antibodies reduced the entry of KSHV, inhibition by β1 integrin antibodies could also be blocking virus interactions with α3β1 integrin. Studies demonstrate that the localization of these integrins in the host cell lipid raft of HMVEC-d cells is important for KSHV infection. KSHV-dependent clustering of integrins α3β1, αVβ3, αVβ5 and CD98 has been reported and the initial binding of KSHV to the cell surface is suggested to be via αVβ3 [66]. Further studies are needed to clarify this suggestion.

Collectively, these studies demonstrate that KSHV infection involves complex virus–host cell surface interactions leading to formation of a multi-molecular complex of integrins that play roles in infection [30].

### 3.4. Role of xCT and DC-SIGN Molecules in KSHV Infection

Host cell surface membrane glycoprotein CD98 associated 12 transmembrane glutamate/cysteine exchange transporter xCT molecule has been identified as a potential KSHV fusion-entry receptor [68]. It is interesting to note that CD98 has been shown to regulate the various cellular processes such as integrin activation, amino-acid transport, cell adhesion, and cell proliferation, and CD98 interacts with integrin α3 to regulate fusion between cells and cell fusion by viruses [58,69,70]. The xCT molecule has been identified in the KSHV infection induced multimolecular signaling complex formed during macropinocytosis of virus in HMVEC-d cells and pretreatment of KSHV with heparin and soluble α3β1 integrin inhibits α3β1-xCT complex formation during infection [30,45]. Further studies are essential to identify the envelope glycoproteins of KSHV mediating the interactions with xCT.

Several viruses, such as human immunodeficiency virus (HIV), hepatitis C virus, Dengue virus and Bunyaviruses, utilize DC-SIGN on the dendritic cell (DC) surface [71,72,73,74]. Activated macrophages and B cells express DC-SIGN while the endothelial cells express its isomer, DC-SIGNR [23,60,75]. During infection of human myeloid DCs, macrophages, and activated B cells, KSHV has been shown to utilize DC-SIGN. Pretreatment of cells with anti-DC-SIGN antibodies or with mannan, the natural ligand of DC-SIGN, as well as pretreatment of KSHV with soluble DC-SIGN has been shown to inhibit virus binding and infection [23,60]. Due to increased expression of DC-SIGN, B cells are suggested to be more susceptible to infection; however, only a partial block of KSHV infection by anti-DC-SIGN antibodies suggests the role of additional binding receptors such as HS and/or other receptors in cells in which DC-SIGN is used as binding and entry receptor. In addition to the integrins (α3β1, αVβ3, and αVβ5), DC-SIGN has also been reported to be a KSHV entry receptor in the human monocytic cell line THP-1 since blocking DC-SIGN reduces KSHV entry without affecting virus binding to these cells [57] (Table 1). A recent study also suggests the role played by DC-SIGN as KSHV receptor in DC cells, as inhibition of viral interaction with DC-SIGN strongly reduced KSHV entry as well as ~60% reduction in KSHV-mediated STAT3 phosphorylation [76].

### 3.5. Role of Ephrin Type-A Receptor 2 (EphA2R) in KSHV Infection

EphA2R, a tyrosine kinase contributing to neo-vascularization and oncogenesis, plays a pivotal role in KSHV infection of cells of endothelial and fibroblast origin. The receptor has been implicated as a center for signaling events and control of macropinocytosis and clathrin dependent endocytosis in different cells [77,78]. Use of soluble EphA2 ligand or preincubation of KSHV virions with soluble EphA2, EphA2R knockdown and overexpression studies have revealed that KSHV glycoprotein gH and gL interacts with EphA2R to gain entry into the cell (Table 1). The gH/gL binding with EphA2R induces EphA2R phosphorylation and internalization of the virus, thus suggesting that EphA2R is a specific cellular receptor for KSHV [61].

Studies highlight the important role of EphA2R during KSHV macropinocytosis in HMVEC-d endothelial cells. KSHV binding and interaction with HS, various integrins and xCT first occurs in the non-lipid raft (NLR) region. This interaction is followed by infection induced c-Cbl mediated rapid translocation of KSHV along with α3β1 and αVβ3 integrins and xCT receptors to the LR region. KSHV interacts with the LR associated EphA2R which then associates with and aids in the formation of the active signaling complex between the integrins, c-Cbl and myosin IIA to induce the formation of macropinocytic membrane blebs. EphA2R also binds to several signaling molecules, including FAK, Src, and the c-Cbl-myosin IIA complex in the LRs to allow the retraction of membrane blebs and macropinocytosis of KSHV into early macropinosomes which subsequently traffic towards the nucleus for a productive infection [79]. EphA2R has also been shown to play a crucial role in KSHV entry through clathrin mediated endocytosis in HFF cells [36].

## 4. KSHV Interactions with Cell Surface Receptors Induce a Cascade of Host Preexisting Signal Pathways That Aid in Virus Entry and Trafficking

### 4.1. Early during Infection, KSHV Induces FAK, Src, PI3-K and Rho-GTPase to Facilitate Its Entry and Infection

The interaction of KSHV glycoproteins with host cell binding and entry receptors triggers the induction of intracellular tyrosine kinases to induce internalization which is supported by the fact that inhibition of tyrosine phosphorylation blocked virus entry but not virus binding [19,20]. KSHV interactions with the integrins induce autophosphorylation of Focal adhesion kinase (FAK) which then interacts with downstream molecules such as Src, PI3-K and c-Cbl to modulate trafficking (Figure 1).

FAK (focal adhesion kinase) is a multi-domain non-receptor tyrosine kinase protein which upon activation by ligand–integrin interaction induces several host cell processes such as adhesion, proliferation, migration, endocytosis and apoptosis [80,81]. KSHV induces the signaling cascade within minutes of infection by the autophosphorylation of FAK at tyrosine 397, a major phosphorylation site required for the outside-in signaling of integrins [80,82]. This FAK induction has been demonstrated in KSHV infected HMVEC-d, HFF, HEK293, and FAK^+/+^ mouse DU17 fibroblast cells [43,44,47,63,64,83]. Purified KSHV gB also induces FAK autophosphorylation, and within minutes of infection, KSHV-induced FAK colocalizes with cytoskeleton associated vinculin and paxillin proteins as well as Src, PI3-K and RhoA-GTPase signal molecules in the infected cells [43,47].

Studies on FAK^+/+^ mouse DU17 fibroblasts and FAK^−/−^ DU3 cells show that phosphorylation of FAK and FAK induced signaling are important for KSHV entry [64,62] (Table 1). Although similar levels of KSHV binds to FAK^−/−^ DU3 and FAK^+/+^ DU17, internalization of KSHV DNA is reduced by >70% in FAK^−/−^ DU3 cells, and FAK expression in FAK^−/−^ DU3 augments viral DNA internalization. In contrast, expression of FAK dominant negative mutant FAK-related nonkinase (FRNK) in FAK^+/+^ DU17 cells results in significant reduction in KSHV entry. KSHV infection induces the FAK related Pyk2 phosphorylation and the Pyk2 molecule is known to compensate some of the functions of FAK [61]. However, KSHV enters the Pyk2 positive FAK^−/−^ DU3 cells in much lower levels and viral entry is reduced in DU3 cells expressing the autophosphorylation mutant of Pyk2 [61]. These studies demonstrate that as FAK activation is vital for many processes such as outside-in signaling, actin modulation, and endocytosis, KSHV has evolved to take advantage of the signaling cascades initiated by FAK to facilitate it entry and infection.

Induction of FAK and Pyk2 in turn results in the activation of Src kinases such as the Src, Lyn, Fyn, Yes, Lck, Blk, and Hck leading into the activation of various signal pathways including PI3-K and Rho-GTPases. The autophosphorylation site of FAK (Tyr 397) creates a binding site for the SH2 domain of Src kinases, and the phosphorylated Src colocalizes with FAK and induces a variety of intracellular signaling by phosphorylating PI3-K and other downstream targets such as Rho-GTPases [63]. KSHV infection induces a vigorous Src response within minutes and colocalizes with FAK. Src activation is required for the regulated entry of KSHV in target cells as KSHV fails to enter Src-negative mouse fibroblast cells and LR disruption increases Src kinase activity and KSHV entry [83]. The coordinated activities of Src and RhoA, and feedback activation of Src by RhoA is also required for the internalization of KSHV in the epithelial HEK293 cells [63].

The membrane associated PI3-K is a heterodimeric protein that consists of the p85 regulatory subunit and the catalytic p110 subunit, and activation of PI3-K by FAK/SRC and other pathways results in the phosphorylation of p85 at the tyrosine residues. PI3-K activation is observed within the first 5 min of KSHV infection which decreases after 15 min p.i. [64]. Inhibition of PI3-K by wortmannin and LY294002 blocked virus entry which demonstrates that KSHV induces PI3-K to facilitate its entry into the target cells [64]. PI3-K p85 phosphorylation induction by KSHV-gB is blocked by preincubation of the protein with heparin as well as by the inhibition of Src with SU6656 while the induction of Src phosphorylation by KSHV infection is not inhibited by the PI3-K inhibitors. These results demonstrate that KSHV induced Src activation is upstream to the induction of PI3-K [47]. Absence of PI3-K induction in FAK negative DU3 cells by KSHV and activation of p85 in FAK positive DU17 cells demonstrate that FAK and Src play critical roles in PI3-K induction during infection and activation of FAK, Src and PI3-K are all needed for virus entry.

KSHV induction of PI3-K in turn initiates the activation of Rho-GTPases and downstream effector molecules as well as the adaptor cCbl protein all of which play roles in the endosome formation and endosome trafficking of KSHV [63]. Rho-GTPase family RhoA, Rac, and Cdc42 proteins regulate signal pathways that control the modulation of cytoskeleton, actin and membrane dynamics [80,84,85]. KSHV infection and addition of purified KSHV gB induce the cytoskeletal rearrangement in the target cells that is dependent upon the induction of the FAK-Src-PI3-K-Rho-GTPase cascade which results in the formation of actin dependent lamellipodia, filopodia and stress fibers in the cells [24,63,64,86]. Expression of dominant-negative RhoA molecule and inhibition of RhoA by *Clostridium difficile* toxin B (CdTxB) results in inhibition of KSHV entry [63]. A sustained feedback activation of Src and the regulation of KSHV endocytosis depends upon the infection induced RhoA and Dia-2 (a formin family member) molecules [63].

### 4.2. Early during Infection, KSHV Induces c-Cbl, CIB1, EphA2R, Cas, and Crk to Facilitate Its Entry and Trafficking

c-Cbl is a multifunctional adaptor protein with E3 ubiquitin ligase activity that regulates the signal pathways by ubiquitinating target proteins to govern their cellular localization, phosphorylation, and interaction with other signal molecules [87,88]. c-Cbl has been shown to regulate KSHV target cell infection and the role of c-Cbl’s in promoting macropinocytosis was reported for the first time during KSHV macropinocytic entry in HMVEC-d cells [29,30]. In a PI3-K dependent manner, c-Cbl tyrosine phosphorylation is induced by KSHV as early as 1 min p.i. in HMVEC-d cells, which is needed for bleb formation, actin and myosin-IIA dependent plasma membrane protrusions, as well as for the bleb mediated macropinocytosis of KSHV [88]. As early as 5 min p.i., KSHV infection induced the recruitment of activated c-Cbl and myosin IIA to the bleb regions [29]. c-Cbl-myosin IIA interaction and c-Cbl mediated myosin IIA ubiquitination is essential for bleb mediated macropinocytosis of KSHV in HMVEC-d cells as knockdown of c-Cbl results in the inhibition of virus entry by macropinocytosis [29].

Soon after KSHV binding to the HS and integrins, infection induced PI3-K activates c-Cbl, which in turn mediates differential ubiquitination of viral entry receptor to regulate the virus entry pathways and their fate [30]. In HMVEC-d and HUVEC cells, the c-Cbl mediated ubiquitination of KSHV entry receptor β1 integrins has been shown to initiate viral particle internalization [30,89]. Other studies show that c-Cbl selectively monoubiquitinates KSHV entry receptors integrin β1 and β3 molecules to facilitate KSHV macropinocytosis in HMVEC-d cells leading towards a successful infection whereas it polyubiquitinates integrin β5 to direct clathrin mediated KSHV endocytosis and for directing KSHV towards lysosomal degradative pathways [30]. In HFF cells, c-Cbl gets engaged with EphA2R to facilitate polyubiquitination (K63 type) of the EphA2R to promote clathrin mediated endocytosis of KSHV as siRNA against c-Cbl inhibits KSHV association with clathrin and the EphA2 receptor [79]. CIB1 (Calcium and integrin binding protein-1), a 22-kDa ubiquitously expressed protein, amplifies the EphA2R associated signaling and promotes KSHV macropinocytosis [31]. Knockdown of CIB1 results in a significant reduction in KSHV-induced bleb formation, activation of EphA2R, Src, and ERK1/2, macropinocytosis of virus particles, endosome trafficking, and viral gene expression [31]. CIB1 plays an important role in scaffolding EphA2R with cytoskeletal myosin IIA and alpha-actinin 4 during KSHV entry [31]. A significant increase in KSHV entry in HEK293 cells overexpressing CIB1 correlated with the reduction in KSHV entry by ~70% in CIB1 knockdown HMVEC-d cells. Studies have shown that CIB1 is an enhancer of FAK, ERK1/2, and PAK1 kinase actions [90,91], and calcium is an important divalent cation that regulates membrane blebbing, integrin signaling and vesicular trafficking [92]. KSHV is also known to induce calcium immediately (~30 s) via Src induction and Src association with plasma membrane associated L-type calcium channel Cav1.2 after infection in HUVEC cells [93]. Calcium also plays an important role in herpes simplex virus and Coxsackie virus entry associated signaling events [94,95]. Further studies are essential to decipher the potential role of calcium influx during CIB1 mediated cell signaling which may further enhance understanding of the complex KSHV entry process in host cells.

Upon its activation (phosphorylation), host cell scaffold docking p130Cas protein associates with several adaptor-effector complexes. *Yersinia pseudotuberculosis* protein invasin interacts with cell surface β1 integrin to induce FAK and Src that recruits p130Cas and Crk which in turn activates Rac leading to actin-mediated phagocytosis [96]. During adeno virus infection, αVβ5 integrin-PI3-K-p130cas has been shown to facilitate viral endocytosis [97]. Studies demonstrate that p130Cas plays a critical role during KSHV infection in HMVEC-d cells [65]. KSHV integrin α3β1 and αVβ3 interaction induced signal molecules promote the recruitment of CIB1, p130Cas, and Crk molecules to the integrin-KSHV interacting sites on the NLR regions of the plasma membrane. Virus interaction with EphA2R in the LR region induces the association of EphA2R with CIB1 resulting in the phosphorylation of p130Cas, assembling of the EphA2R-CIB1-c-Cbl-Crk signalosome which in turn activates the GEF-C3G (guanine nucleotide exchange factor phospho-C3G) molecule which probably directs the GTPase signaling to accelerate the KSHV containing macropinosome trafficking (Figure 1). Live cell imaging of KSHV infection of control and p130Cas knockdown HMVEC-d cells show that in the absence of p130Cas, KSHV is directed toward lysosomal degradation and thus demonstrating the critical role of p130Cas in KSHV infection [65].

## 5. Lipid Rafts (LR) of Infected Cells Regulate KSHV Entry and Trafficking

LRs are dynamic assemblies of sphingolipids (sphingomyelin and glycosphingolipids) and cholesterol in the outer leaflet of the plasma membrane involved in major signaling events by promoting receptor clustering and various protein-protein and protein-lipid interactions [98]. Studies show that the LRs play a critical role in KSHV de novo infection. KSHV induced activated c-Cbl induces the selective translocation of KSHV along with α3β1 and αVβ3 integrins and xCT receptors into the LR region of the plasma membrane to associate and activate the entry receptor, EphA2R, resulting in the enhancement of EphA2 kinase action that amplifies the downstream signals [30,79]. Simultaneously, CIB1 is also translocated to the LR to aid the EphA2R initiated signal amplification and sustains EphA2R phosphorylation, and finally associating with Src, c-Cbl, PI3-K, alpha-actinin 4, and myosin IIA to enhance EphA2R crosstalk with the cytoskeleton to recruit macropinosome complex formation [31]. Disruption of LRs by methyl-beta-cyclodextrin (MβCD) or nystatin did not affect KSHV binding but resulted in an increase in KSHV entry, a significant reduction in the nuclear entry of KSHV genome and therefore reduced viral gene expression. Further studies revealed that disruption of LRs increases the induction of p-Src by KSHV without affecting FAK or ERK1/2 activation but greatly reduces the activation of PI3-K, Rho-GTPase, and NF-κB, and subsequently abolishing RhoA mediated acetylation and microtubule aggregation which are important events during entry stages of infection, trafficking of KSHV in cytoplasm and nuclear delivery of viral genome [83]. These studies together demonstrate the role of LRs during KSHV entry in HMVEC-d cells and suggest that LRs serve as the regulatory hub platform for KSHV interactions with entry receptors and the infection induced signalosome assembly [31].

## 6. KSHV Induces Signal Pathways to Regulate Its Trafficking in the Cytoplasm Early during Infection

### 6.1. KSHV Infection Induces RhoA-GTPase to Modulate the Acetylation of Microtubules to Facilitate Intracellular Capsid Movement

KSHV entry and trafficking into the target cell is a rapid process, as it penetrates the host cytosol to deliver its genome into the nucleus as early as 15 min p.i. which peaks by 90 min p.i. [24,63,64,99]. KSHV utilizes the host cell microtubule network which is tightly associated with Rho-GTPases. RhoA, Rac, and Cdc42 Rho-GTPases regulate a variety of signaling pathways and consequently different cellular processes including cytoskeleton rearrangement and morphological changes. KSHV interactions with cell surface integrins induces PI3-K-Rho-GTPase dependent modulation of actin leading into the formation of filopodia, lamellipodia and stress fibers (RhoA) in HMVEC-d, HUVEC and HFF cells [64,99,100]. Dominant negative RhoA-GTPase expression and pretreatment of cells with a specific inactivator of Rho-GTPases, *Clostridium difficile* toxin B (CdTxB), significantly blocks KSHV entry (Table 1). Studies demonstrating the inhibition of Src by CdTxB and inhibition of RhoA by Src inhibitors suggest that KSHV-induced Src is involved in RhoA activation, which in turn is involved in a feedback sustained activation of Src [64,99].

KSHV infection induces the aggregation and thickening of microtubules (MT) in the infected cells [99]. KSHV capsids colocalize with the microtubules which is eliminated by blocking with PI3-K inhibitor which blocks the Rho-GTPases and by the nocodazole mediated destabilization of microtubules [99]. It is interesting to note that the inactivation of Rho-GTPases by CdTxB also blocked microtubular acetylation and subsequently the delivery of viral DNA to the nucleus. These studies demonstrate that KSHV infection induced Rho-GTPase is involved in microtubule acetylation and aggregation [99]. Expression of the constitutively active RhoA mutant increases the nuclear delivery of KSHV genome and, in contrast, expression of a dominant negative mutant of RhoA results in the decrease of KSHV genome nuclear delivery [99]. Activation of Rho-GTPases by *Escherichia coli* cytotoxic necrotizing factor results in an augmented nuclear delivery of KSHV DNA [99].

KSHV capsid is rapidly transported towards the nucleus on the RhoA-GTPase acetylated microtubules via the dynein proteins. Inhibition of dynein motor proteins by sodium orthovanadate blocked the nuclear delivery of KSHV genome and thus viral gene expression (infection) [99]. RhoA-GTPases activate Diaphanous 1 and 2 molecules leading to rearrangement of the cytoskeleton [99]. KSHV infection induces the activation of Dia-2 via RhoA-GTP and Dia2 co-immunoprecipitates and colocalizes with activated Src in the infected cells, which are inhibited by Src inhibitors [99]. Reduction of KSHV entry in cells expressing dominant negative RhoA demonstrate that activated RhoA-dependent Dia2 acts as a link between RhoA and Src in infected cells and mediates the sustained Src activation and that KSHV-induced Src and RhoA play roles in facilitating not only in virus entry but also in the nuclear delivery of viral DNA via the acetylation of microtublues.

Together with the studies of LR inhibition abolishing the KSHV induced PI3-K and Rho-GTPase activation, RhoA mediated acetylation and microtubule aggregation, nuclear entry of viral genome and viral gene expression demonstrate that KSHV has evolved to induce the integrin-EphA2R-signal pathways not only to modulate endocytic entry, but also to modulate the microtubule acetylation/stabilization and thus to promote the rapid trafficking of viral capsids toward the nucleus [99]. These studies also demonstrate for the first time the modulation of the microtubule dynamics by virus-induced host cell signaling pathways to aid in the trafficking of viral DNA containing capsid.

Confocal microscopy has revealed that the KSHV cargos internalized due to the synergistic effect of EphA2R and CIB1 in HMVEC-d cells colocalizes with Rab5 in the early macropinosome while the shRNA against EphA2R leads to the abolishment of KSHV trafficking into Rab5 positive endosomes. A similar finding has also been reported in HFF cells which confirm the strict requirement of these KSHV trafficking pathways [36].

Another study shows that reactive oxygen species (ROS) is induced by KSHV early during infection via its binding to the cell surface receptors and pretreatment of the virus with soluble heparin abolished ROS induction [101]. The ROS production also plays an important role in the entry of the virus as the use of ROS inhibitor N-acetyl cysteine (NAC) blocked KSHV infection by blocking virus entry, translocation of αVβ3 integrin into lipid rafts, actin-dependent membrane perturbations, membrane bleb formation, and phosphorylation of the EphA2R, FAK, Src, and Rac1, and in contrast, treatment with H_2_O_2_ induced the activation of EphA2R, FAK, Src, and Rac1 [102]. This study demonstrates that KSHV induces ROS to promote its entry and to amplify the initially induced host signal cascade (Figure 1). One of the downstream targets of ROS is nuclear factor E2-related factor 2 (Nrf2), a transcription factor with important anti-oxidative functions. It has been shown that KSHV induces Nrf2 through complex mechanisms involving multiple signal molecules, which is important for its ability to manipulate host and viral genes, creating a microenvironment favorable to KSHV infection [103].

### 6.2. KSHV Utilizes the ESCRT Complex Proteins for Its Entry and Trafficking

The endosomal sorting complexes required for transport (ESCRT) proteins include ESCRT-0, -I, -II, and -III, which together with the VPS4 ATPase function in a sequential manner to mediate the endosomal trafficking and sorting of internalized and ubiquitinated receptors [104]. Since the initial discovery that HIV-1 utilizes the ESCRT pathway to bud from the plasma membrane [105,106], many other viruses have been shown to utilize this pathway for their egress. Apart from viral egress, the role of ESCRT proteins has also been documented in virus entry. For example, a recent publication demonstrates the role of Tsg101 (ESCRT-I component), Vps24 (ESCRT-III component), and Vps4B (ATPase component) during Crimean-Congo hemorrhagic fever virus entry into target cells [107]. Studies on Rota virus [108] and old world arena viruses [109] also reveal the important roles of these proteins in virus entry and infection.

The ESCRT-0 component Hrs is shown to play an important role during KSHV entry in HMVEC-d cells by macropinocytosis [32]. Knockdown of Hrs results in a significant inhibition of KSHV entry and subsequent gene expression in HMVEC-d cells. IFA and proximity ligation assay (PLA) also demonstrate for the first time that Hrs translocates from the cytosol to the plasma membrane of KSHV infected cells where it modulates the local pH change to facilitate KSHV macropinocytosis [32].

A recent study demonstrates that the ESCRT-I complex protein Tsg101 plays an important role during KSHV trafficking. Tsg101 interacts with all the crucial group of signal proteins that are known to be associated with KSHV macropinocytosis and associates with trafficking of the KSHV via Rab5 and Rab7 associated early and late endosomes, respectively [33]. siRNA mediated knockdown reveals that Tsg101 did not affect KSHV entry but significantly inhibits the transition of KSHV from early to late endosomes and consequently significant reduction in the nuclear delivery of KSHV genome leading to a drastic reduction in viral gene expression [33] (Table 1). The fate of the virus and further studies are needed to fully elucidate the role of Tsg101 and other ESCRT complex proteins during KSHV entry and productive infection.

## 7. Early during Infection, KSHV Induces the Signal Pathways to Regulate the Host Transcription Factors NF-κB, ERK1/2 and Nrf2 to Facilitate Infection

KSHV viral genome entry into the nucleus is a rapid process and viral genome enters within 15 min of infection of HMVEC-d and HFF cells [57], and viral gene expression starts between 15 and 30 min p.i. Studies with live and UV-inactivated KSHV suggests the cell surface receptor interaction initiates the induction of ERK1/2 and NF-κB during the early time of infection and KSHV viral gene expression is required for sustained induction [110,111]. To initiate its gene expression as well as modulate various host genes, KSHV utilizes its cell surface receptor interactions to induce a robust level of NF-κB, ERK1/2 and Nrf2 transcription factors. ERK1/2 induction can be observed as early as 5 min p.i. by high and low multiplicity of infection of both live and UV-inactivated KSHV in the absence and presence of fetal calf serum in the cell culture medium, and PI3-K and PKC-ζ are the upstream mediators of ERK1/2 induction pathway [64]. KSHV infection also induces the ERK1/2 regulated host transcription factors c-Jun, STAT1, MEF2, c-Myc, ATF-2, and c-Fos early during infection. MEK inhibitor U0126 inhibits viral gene expression [110] as well as the activation of host c-Fos, c-Jun, c-Myc, and STAT1 molecules.

KSHV also induces NF-κB at 5 min p.i. in HMVEC-d and HFF cells with rapid translocation of p65-NF-κB into the infected cell nuclei which is blocked by Bay11-7082 inhibiting the IκB phosphorylation [111]. Sustained moderate levels of NF-κB induction persist subsequently with p38 MAPK activation occurring at later time points. Inhibition of NF-κB did not affect virus entry but blocks Jun D, Jun B, phospho-c-Jun, cFos, and FosB activation as well as viral gene expression [111]. Studies demonstrate that the nuclear factor E2-related factor 2 (Nrf2), a transcription factor, functions as an important host factor involved in the establishment of de novo KSHV infection. Studies also show that ROS is essential for Nrf2 activation during early stages of KSHV infection of HMVEC-d cells and Nrf2 plays a crucial role in the viral gene expression [103,112]. Nrf2 knockdown or inhibition with the chemical Brusatol blocks viral gene expression [112].

Collectively, these studies demonstrate that KSHV interaction with the cell surface receptors induces ERK1/2, NF-κB and Nrf2 very early during infection for the initiation of viral gene expression and host cell genes to undoubtedly overcome the host cell restrictions on viral gene expression.

## 8. Nuclear Delivery of KSHV dsDNA Genome Induces Host Nuclear Innate Responses

Entry of KSHV’s dsDNA genome into the nucleus of the infected cells elicits host innate responses and we discuss briefly the responses here.

### 8.1. Nuclear DNA Damage Response (DDR) Induction Early during KSHV Infection

The DNA damage response (DDR) is an extensive regulatory signaling mechanism present in all mammalian cells to sense and repair different types of cellular DNA damage [113]. DDR acts as a signal transduction cascade, and lesions in the DNA are detected by the DDR sensor proteins, which in turn activate kinases, leading to amplification of the signals through a series of downstream effector molecules. The host’s repair machinery, in addition to the cellular DNA damage, also recognizes the exogenous genetic material, such as the viral DNA genome entering the nucleus during infections. However, several studies show that many DNA viruses also manipulate the key signaling events of the DDR pathway for their own advantage [114].

KSHV modulates DDR signaling during de novo infection of primary endothelial cells. Viral genome entry into the nuclei, as early as 13–30 min p.i., induces the phosphorylation of DDR-associated proteins such as ataxia telangiectasia mutated (ATM) and H2γX, and H2γX colocalizes with the KSHV genome [115]. Inhibition of ATM kinase activity and siRNA mediated knockdown of H2AX results in >80% reduction in the viral gene expression demonstrating a role for the DDR proteins in the viral life cycle early during infection [115].

### 8.2. Nuclear Innate Immune Response Induction Early during KSHV Infection

Previous studies have shown that cytoplasmic foreign molecules, including pathogens and dsDNA, are recognized by cytoplasmic sensors such as NLRP3 and AIM2, which results in the homotypic sensor interactions with the adaptor protein ASC which in turn interacts with procaspase-1 (the inflammasome platform) to induce caspase-1 activation, cleavage of pro-IL-1β and IL-1β cytokine maturation. Studies with KSHV for the first time demonstrate that the innate sensing resulting in inflammasome formation also occurs in the nucleus. Soon after viral genome entry into the nucleus, KSHV genome is recognized by the interferon gamma-inducible protein 16 (IFI16) [116]. IFI16 is a predominantly nuclear protein involved in transcriptional regulation. Recognition of nuclear replicating episomal KSHV, EBV, and HSV-1 genomes by IFI16 results in the formation of the IFI16-ASC-procaspase-1 inflammasome in the nucleus, which is transported to the cytoplasm leading into caspase-1 activation and pro-IL-1β/IL-18 cleavages [108,109,110]. Independent of ASC, KSHV and HSV-1 genome recognition results in IFI16 interaction with STING in the cytoplasm, phosphorylation and nuclear translocation of IRF3, IFN gene expression and IFN-β production [116]. These studies show that IFI16 acts as the nuclear pathogen sensor.

A recent study demonstrated that the host cell BRCA1 protein, a transcription factor and DNA damage response protein, complexed with IFI16 regulates the nuclear innate sensing of KSHV, EBV and HSV-1 genomes by IFI16 and IFI16-ASC-procaspase-1 inflammasome formation [117]. The study also shows that BRCA1 is responsible for the cytoplasmic IFI16-STING signalosome activation and induction of IFN-β during de novo KSHV infection [117]. A concurrent study also shows that nuclear to cytoplasmic trafficking of IFI16 during herpesvirus infection is Ran-GTP dependent and mediated by the acetylation of IFI16 by the histone acetyl transferase p300. The acetylation of IFI16 is essential for the IFI16-ASC interaction and inflammasome activation. This post-translational modification of IFI16 is also essential for the cytoplasmic association of IFI16 with STING resulting in IRF-3 phosphorylation, nuclear pIRF-3 localization and interferon-β production [118]. Further studies are needed to define how KSHV overcomes these innate nuclear responses.

## 9. Conclusions

KSHV has successfully evolved with a striking survival strategy that reflects the biological complexity of the virus and host interactions. The studies summarized here demonstrate that for a successful infection of endothelial and fibroblast cells, KSHV has evolved to interact with key cell surface molecules which not only initiate its infection but also set the stage: (a) for the induction of various host cell signal pathways to coordinate and regulate the various endocytic mechanisms such as macropinocytosis and clathrin mediated endocytosis to aid in the rapid entry of viral particles into different cell types; (b) to modulate the various host cell functions for its speedy traffic in the dense cytoplasm towards the nucleus, including the efficient use of the host ESCRT machinery to commute through the cytoplasm; and (c) to induce the cytoplasmic ERK1/2, NF-κB and Nrf2 transcription factors very early during infection to initiate viral gene expression soon after the entry of viral genome into the nucleus. Further studies are needed to explore the viral strategies to overcome the host innate immune response molecules during entry and nuclear delivery of the viral genome. As these studies demonstrate that KSHV entry and infection are due to the combined effects of various receptors, associated signaling, cytoskeletal proteins and adaptor molecules, these provide a clear framework for further exploration of combinations of drugs and/or small molecules that can simultaneously target the receptors and the signal molecules to inhibit the target cell infection by KSHV.

## Figures and Tables

**Table 1:** Summary of cell surface receptors utilized by KSHV and induced signal molecules, the methods used to block them and their effects on KSHV entry and infection.

**KSHV Receptors**	**Inhibitor/Treatment**	**Cell Type**	**Effect**	**References**
Heparan sulfate	Pre-incubation of virus with soluble heparin	HMVEC-d, HFF, BJAB, HEK293, THP1	Blocks virus binding to the cell	[21,41,43,44,45,51,54,55,57,58]
α3β1 integrin	Pre-treatment of cells with anti- α3β1 antibodies; Pre-incubation of virus with soluble α3β1 integrin	HMVEC-d, HFF, THP1	Blocks virus entry and no effect on virus binding	[44,45,57]
αVβ3 integrin	Pre-treatment of cells with anti-αVβ3 antibodies; Pre-incubation of virus with soluble αVβ3 integrin	HMVEC-d, HFF, THP-1	Blocks virus entry and no effect on virus binding	[45,57,59]
αVβ5 integrin	Pre-treatment of cells with anti-αVβ5 antibodies; Pre-incubation of virus with soluble αVβ5 integrin	HMVEC-d, HFF, THP-1	Blocks virus entry and no effect on virus binding	[44,59]
xCT	Pre-treatment of cells with anti-xCT antibodies	HMVEC-d	Blocks virus gene expression and no effect on virus binding and entry	[44]
CD98	Pre-treatment of cells with anti-CD98 antibodies	HMVEC-d	Blocks virus gene expression and no effect on virus binding and entry	[44]
DC-SIGN	Pre-treatment of cells with Mannan; Pre-treatment of cells with anti-DC-SIGN antibodies	B cells, THP-1	Blocks virus binding and entry	[23,57,60]
EphA2R	Pre-treatment of cells with anti-EphA2R antibodies; Pre-incubation of virus with soluble EphA2	HMVEC-d, HFF	Blocks virus entry and no effect on virus binding	[35,61]
**Signal Molecules Induced by KSHV**	**Inhibitor/Treatment**	**Cell Type**	**Effect**	**References**
FAK	Focal Adhesion Kinase (FAK)-related Non-kinase (FRANK)	DU17 mouse embryonic fibroblasts	Blocks virus entry	[62]
Src	SU6656	HEK293	Blocks virus entry	[63]
PI3K	LY294002	HMVEC-d, HFF	Blocks virus entry	[64]
RhoA	Clostridium difficile toxin B	HMVEC-d, HFF	Blocks virus entry	[63]
PKCζ	myr-ζ	HFF	Blocks viral gene expression	[64]
CIB1	shCIB1	HMVEC-d	Blocks virus entry	[31]
p130Cas	shCas	HMVEC-d	Blocks nuclear viral genome delivery	[65]
Hrs	shHrs	HMVEC-d	Blocks virus entry	[32]
Tsg101	siTsg101	HMVEC-d	Blocks cytoplasmic KSHV trafficking and nuclear viral genome delivery	[33]
